# Validation of formulae predicting stroke volume from arterial pressure: with particular emphasis on upright individuals in hot ambient conditions

**DOI:** 10.3389/fphys.2024.1398816

**Published:** 2024-07-10

**Authors:** Lydia Tsoutsoubi, Leonidas G. Ioannou, Urša Ciuha, Jason T. Fisher, Carmen Possnig, Lydia L. Simpson, Andreas D. Flouris, Justin Lawley, Igor B. Mekjavic

**Affiliations:** ^1^ Department of Automatics, Biocybernetics and Robotics, Jožef Stefan Institute, Ljubljana, Slovenia; ^2^ International Postgraduate School Józef Stefan, Ljubljana, Slovenia; ^3^ Department Sport Science, University of Innsbruck, Innsbruck, Austria; ^4^ FAME Laboratory, Department of Physical Education and Sport Science, University of Thessaly, Trikala, Greece

**Keywords:** heat, cardiac index, blood pressure, heart rate, cardiac output, heatwave

## Abstract

**Introduction:**

During heatwaves, it is important to monitor workers’ cardiovascular health since 35% of those working in hot environments experience symptoms of heat strain. Wearable technology has been popularized for monitoring heart rate (HR) during recreational activities, but it can also be used to monitor occupational heat strain based on core and skin temperatures and HR. To our knowledge, no devices estimate the cardiovascular strain directly based on stroke volume (SV) or cardiac output (CO). In addition to the hardware, there are limitations regarding the lack of suitable algorithms that would provide such an index based on relevant physiological responses. The validation of the formulae already existing in literature was the principle aim of the present study.

**Methods:**

We monitored the cardiovascular responses of our participants to a supine and 60° head-up tilt at the same time each day. During the test, we measured blood pressure derived by finger photoplethysmography, which also provided beat-by-beat measures of SV and CO. Afterwards, we compared the SV derived from the photoplethysmography with the one calculated with the different equations that already exist in literature.

**Results:**

The evaluation of the formulae was based on comparing the error of prediction. This residual analysis compared the sum of the squared residuals generated by each formula using the same data set.

**Conclusion:**

Our findings suggest that estimating SV with existing formulae is feasible, showing a good correlation and a relatively small bias. Thus, simply measuring workers’ blood pressure during breaks could estimate their cardiac strain.

## 1 Introduction

Having evolved in the equatorial region, humans are considered tropical animals (Kenney et al., 2014). This evolutionary advantage provides an efficient concerted action of the thermoregulatory and cardiovascular systems in maintaining body temperature within narrow limits (Kenney et al., 2014; Crandall and Wilson, 2015; Flouris and Piantoni, 2015) and ensures appropriate arterial pressure for adequate perfusion of vital organs during exposure to hot ambient conditions in the upright posture (Crandall and Wilson, 2015). Exposure to hot ambient conditions causes an immediate and sustained vasodilatation, which must be matched by an increase in cardiac output (CO) through augmented heart rate (HR) and cardiac contractility (Crandall and Wilson, 2015) to ensure maintenance of arterial pressure. Thus, in addition to the thermoregulatory actions, cardiovascular regulation plays a key role in the process of heat acclimatization that enables healthy individuals to work relatively unhindered in hot environments. For a healthy individual in the upright position, every degree Celsius increase in body core temperature results in an increase in heart rate of approximately 33 beats per minute, a response referred to as thermal cardiac reactivity (Ioannou et al., 2021; Ioannou et al., 2019; ISO 9886, 2004). Such increases in HR induced by elevations in core temperature are unrelated to the increases caused by physical labor in a hot environment.

Monitoring cardiovascular health during heatwaves is particularly important for workers, as 35% of individuals exposed to occupational heat stress experience symptoms of occupational heat strain (Flouris et al., 2018). By definition, heatwaves increase both daytime and nighttime ambient temperatures, thus preventing appropriate recovery from the heat strain encountered during work (Ciuha et al., 2019; Ioannou et al., 2021). A proper assessment of the cardiac strain imposed by heatwave-induced changes in the ambient conditions would require monitoring workers regularly throughout the day for the duration of the heatwave. Monitoring occupational heat strain is of paramount importance during summer months since climate change has caused an increase in the frequency, intensity, and duration of heatwaves (Perkins et al., 2012), making it even more difficult for people with underlying cardiovascular diseases to work and survive. Indeed, in such a population, physiological heat strain may have serious health consequences (Kenney et al., 2014). Despite several epidemiological studies (Basu and Samet, 2002; Gasparrini et al., 2015; Amorim et al., 2017; Ioannou et al., 2018; Tsoutsoubi et al., 2021) investigating this issue, the increased cardiovascular morbidity and mortality, particularly in working populations, remains unresolved.

The advances in wearable technology have been popularized for monitoring HR during recreational activities, but some wearables have been used to monitor occupational heat strain (Notley et al., 2018) on the basis of core and skin temperatures, as well as HR (Notley et al., 2018). Where these variables undoubtedly provide an indication of heat strain, particularly on cardiac function, they do so indirectly. To our knowledge, there are no devices that estimate the cardiovascular strain directly, based on stroke volume (SV) and/or CO. In addition to the hardware, there are also limitations regarding the available software or rather the lack of suitable algorithms that would provide such an index based on relevant physiological responses.

Cardiac strain reflects physiological heat strain and can be estimated by the cardiac index, or stroke volume index, defined as the ratio of CO, a product of SV and HR (Plociennik et al., 2017), to the body surface area (Cattermole et al., 2017). Determination of SV normally requires complex clinical methods such as echocardiography, oscillometry, computed tomography, magnetic resonance imaging, and Doppler ultrasound (van Ooijen et al., 2012; Phillips et al., 2017). The measurement of SV using these clinical approaches, and the consequent determination of CO is therefore not practical in an industrial setting.

The measurement of cardiac index in an industrial environment, particularly during tasks that are conducted in hot environments and/or during heatwaves would be of great benefit to workers. In the absence of available wearable technology, arterial blood pressure (ABP) could be measured at regular intervals (i.e., during breaks) using an automated sphygmomanometer and the SV predicted from the measurements of ABP. A major obstacle to the use of such formulae for predicting SV is that they were developed using ABP and HR responses of participants resting in a supine in a, most likely, thermoneutral environment. To be applicable for use in an industrial setting, these formulae need to be validated during acute and prolonged exposures to hot environments, and in individuals maintaining a seated and standing posture during assembly line work. The validation of these formulae was the principle aim of the present study.

## 2 Methods

### 2.1 Formulae evaluated

The formulae predicting SV that were evaluated in the present study are presented in Table 1. The majority of the formulae were derived by Starr and others (Starr, 1954; Starr and Schnabel, 1954), who utilised several experimental approaches to obtain the data necessary to develop these models. The experiments involved, among others, a cadaver experimental model, and studies involving participants with various clinical conditions (Cathcart et al., 1953; Starr, 1954) and participants exposed to different O_2_/N_2_ gas mixtures at atmospheric pressure (Grollman, 1930; Starr, 1954). Furthermore, another study by Jackson created a nomogram for the calculation of SV based on the work of Starr and his colleague (Starr and Schnabel, 1953; Jackson, 1955). The consensus from these studies was that SV could be adequately predicted in resting individuals from diastolic arterial pressure (DAP), pulse pressure (PP = systolic pressure (SAP) - DAP), and age. As evident from Table 1, Starr and others proposed several equations that would predict SV from these variables. Common to these predictive equations is that they were derived from supine and/or seated resting individuals exposed to thermoneutral ambient conditions, and have not been verified in upright and active individuals during exposure to a heat stress. The latter reflecting the common scenario in an industrial environment.

**TABLE 1:** Rating of the formulas that estimate stroke volume based on the absolute values of the correlation, effect size (g), the sum of squared residuals (SSR) and the bias Ranking.

	Author, year (equation)	Equation	r	g	SSR	Bias	Total score
1	Starr 1954 (equation 72) (Starr and Schnabel, 1954)	SV=100+0.5PP−0.6DP−0.6age	0.65	0.96	6,256.11	0.67	15
2	Starr 1954 (equation 71) (Starr and Schnabel, 1954)	SV=101+0.5PP−0.59DP−0.61age	0.65	0.82	6615.34	2.15	18
3	Jackson 1955 (Jackson, 1955)	SV=101+0.5SP−1.09DP−0.61age	0.65	0.82	6,615.34	2.15	18
4	Data from Grollman 1930 (acetylene method) (Grollman, 1930; Starr, 1954)	$SV=0.45\left(93+0.54PP-0.47DP-0.61age\right)+43$	0.63	0.23	8,824.08	3.70	24
5	de Simone 1997 Weight (de Simone et al., 1997)	$SV=3.59\left(weight\right)$ ^0.71^	0.03	0.05	16,487.15	2.01	29
6	de Simone 1997 BSA (de Simone et al., 1997)	$SV=35.38\left(BSA\right)$ ^1.19^	0.00	0.11	16,191.70	1.44	30
7	Starr 1954 (equation 59a) (Starr, 1954)	SV=91+0.54PP−0.57DP−0.61age	0.64	1.39	7,640.52	4.00	30
8	de Simone 1997 Height (de Simone et al., 1997)	$SV=23.99\left(height\right)$ ^2.04^	0.02	0.34	17,122.96	0.01	31
9	Starr 1954 (equation 68) (Starr, 1954; Starr et al., 1955)	SV=93+0.54PP−0.47DP−0.61age	0.63	0.52	8,451.11	4.95	32
10	Starr 1954 (equation 59b) (Starr and Schnabel, 1954)	SV=90.97+0.54PP−0.57DP−0.61age	0.64	1.39	7,660.98	4.03	33
11	Starr 1954 (equation 62) (Starr and Schnabel, 1954)	$SV=90.97+0.73PP-0.57\left(DBP+1/3PP\right)-0.61age$	0.64	1.39	7,660.98	4.03	34
12	Starr 1954 (equation 76) (Starr, 1954)	SV=93+0.62PP−0.45DP−0.61age	0.62	0.03	17,305.87	11.26	35
13	Bridwell 1956 (Bridwell et al., 1956)	SV=66+0.34PP−0.11DP−0.36age	0.57	1.25	9,308.35	3.97	37
14	Starr 1954 (equation 64) (Starr and Schnabel, 1954)	$SV=90.96+0.92PP-0.57\left(DP+2/3PP\right)-0.61age$	0.64	1.40	7,667.83	4.04	37
15	Starr 1954 (equation 63) (Starr and Schnabel, 1954)	$SV=90.97+0.82PP-0.57\left(DP+\frac{1}{2PP}\right)-0.61age$	0.64	1.42	7,871.71	4.33	39
16	Lu 2015 (Lu et al., 2015)	$SV=\left(6.963+0.446-\left(0.037age\right)+\left(0.013weight\right)\right)/HR$	0.58	0.78	59,891.97	20.10	46
17	Skrabal 2005 Weight (Skrabal et al., 2005)	$SV=\left(0.77weight\right)+29$	0.15	1.27	46,574.33	12.22	5
18	Remington 1948(26)	$SV=\left(Volume\;factor\;for\;SBP\;-Volume\;factor\;for\;DBP\right)BSA$	0.42	0.90	128,008.92	35.78	55
19	Data from Warren 1945 (Fick method) (Warren et al., 1945; Starr, 1954)	$SV=1.5\left(91+0.54PP-0.57DP-0.61age\right)+8.9$	0.64	1.67	148,432.03	40.65	61
20	Data from Liljestrand and Zander 1928 (Nitrous oxide method) (Liljestrand and Zander, 1928; Starr, 1954)	$SV=1.81\;\left(93+0.54PP-0.47DP-0.61age\right)-25.9$	0.63	1.67	175,297.23	44.21	63
21	Skrabal 2005 Height (Skrabal et al., 2005)	$SV=\left(1.19height\right)-112$	0.04	3.02	77,434.34	23.31	65
22	Data from Cathcart 1953 (Fick method) (Cathcart et al., 1953; Starr, 1954)	$\text{SV}=1.02\left(93+0.54PP-0.47DP-0.61age\right)+39.6$	0.63	3.40	187,452.27	46.16	68
Ranking order		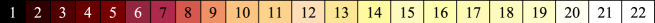					

Note: PP, pulse pressure (mmHg); SP, systolic pressure (mmHg); DP, diastolic pressure (mmHg); age, chronological age (years); Ranking 1 to 21 (1 the best, 21 the worst); bias, mean differences; Total score, the sum of the ranking for each variable from the best to the worst.

Other formulae are based on anatomical measurements, or include anthropometric measurements. An example of the former is the study by Remington and others which measured the length of the aorta during autopsies, and incorporated these measurements in the prediction of stroke volume index from pulse pressure (Remington et al., 1948). Furthermore, a later study adapted one of Starr’s equations to their dataset (Bridwell et al., 1956). More recent research has focused on using solely anthropometric data (such as body weight, height, body surface area, or age) to devise equations that predict either SV or CO (de Simone et al., 1997; Skrabal et al., 2005; Lu et al., 2015) (Table 2).

**TABLE 2:** Variables incorporated in formulae predicting stroke volume.

Author(s)	Variables included in formulae for predicting SV
Starr (Starr, 1954; Starr and Schnabel, 1954), Bridwell (Bridwell et al., 1956), Jackson (Jackson, 1955)	SV ∝ fun (PP, DAP, Age)
Remington (Remington et al., 1948)	SV ∝ fun (Volume Factor for SAP - Volume Factor for DAP)
De Simone (de Simone et al., 1997), Skrabal (Skrabal et al., 2005)	SV ∝ fun (Weight, Height, BSA)
Lu (Lu et al., 2015)	SV ∝ fun (Age, Weight, HR)

Note: SV, stroke volume; PP, pulse pressure; DAP, diastolic pressure; BSA, body surface area; HR, heart rate.

### 2.2 Empirical data

The empirical data used to evaluate formulae predicting SV were derived from a study in which young healthy male participants participated in a simulation of a 3-day heatwave during which they were exposed to day time temperatures of 35.4°C and night time temperatures of 26.3°C. For the purpose of this study, we simulated the intensity of a well-known heatwave that occurred in Paris in 2003, which was responsible for an estimated ∼15,000 heat-related deaths. A heatwave of similar magnitude, but shorter in duration (3 days) was demonstrated. During this heatwave, ambient temperature increased by 9°C compared to the days before and after the heatwave (Ioannou et al., 2021).

The detailed methodology of this study has been reported previously by Ioannou et al. (Ioannou et al., 2021). Briefly, the heatwave was simulated by controlling the temperature and humidity of a simulated workplace and living quarters during a 10-day period. The heat strain experienced by an individual is significantly influenced by their acclimatization status. Hence, the experiments were carried out in the autumn to verify that our participants had not already acclimatized before the study. For this simulation, healthy male participants (n = 7) were confined 24/7 to the Olympic Sport Centre Planica (Rateče, Slovenia) for 10 days. Following a familiarization day, during which anthropometric data were collected, all participants were familiarized with the equipment and study protocol. Participants were required to maintain a 10-day work/rest regimen mimicking that of workers in the manufacturing industry. During the first three (neutral temperature) pre-heatwave (days 1–3) and last three (neutral temperature) post-heatwave (days 7–9) days, the ambient temperature was maintained at 25.4°C (relative humidity, 45%), during the 9-h work shift and at 22.3°C during the remaining 15 h of the day. During the heatwave days (days 4–6) the ambient temperature was maintained at 35.4°C during the work shift and at 26.3°C during the remaining part of the day.

For each participant, we monitored their cardiovascular response to a 60° head-up tilt (HUT) at the same time each day. Following a 10-min period in the supine position, participants were passively tilted to a 60° HUT position for a further 10 min. During the test, we measured blood pressure derived by finger photoplethysmography (Finapres Nova; Finapres Medical Systems BV, Amsterdam, Netherlands), which also provided beat-by-beat measures of SV and CO using the Model Flow algorithm, which is a statistical model of the human circulation, used to compute hemodynamic parameters from the arterial pressure waveform of the finger. Finapres values were calibrated against the brachial artery blood pressure taken at baseline.

### 2.3 Participants

The minimum required sample size for investigating “Differences between two dependent means (matched pairs)” was calculated using the results of a previous study (Shibasaki et al., 2011), which assessed SV on two different occasions in normothermic participants and in individuals under heat stress using different methods. Specifically, we calculated the effect size (dz) for the comparisons between the thermodilution and Finometer methods for the heat-stressed individuals. The calculation was for an effect size (dz) equal to 1.63 for the comparison of SV measured via thermodilution (123 ± 28 mL) versus the Finometer (87 ± 14 mL). Sample size calculations were conducted using G*Power 3.1.9.4 (Faul et al., 2007), setting statistical power and α error probabilities at 0.95 and 0.05, respectively. Based on that, a total of seven healthy male individuals participated in the study (age 21.5 ± 1.2 years; height 180 ± 5.6 cm; weight 81.5 ± 14.5 kg; BSA 2.0 ± 0.2 m^2^; BMI 25.1 ± 4.0 kg/m^2^). Written informed consent was obtained from all volunteers after a detailed explanation of all the procedures involved. The experimental protocol was approved by the National Committee for Medical Ethics of the Republic of Slovenia (no. 0120-402/2020/4) in accordance with the Declaration of Helsinki.

### 2.4 Analysis of the predictive equations

Using the supine and HUT data of participants (n = 7) we validated the predictive power of the formulae presented in Table 1. To validate the formulae we conducted a residual analysis as suggested by Mekjavic and Morrison (Mekjavic and Morrison, 1986), whereby the error of prediction (observed–predicted) was used to derive the sum of the squared residuals (SSR) for each formula. The formulae were then compared on the basis of the SSR. Pearson’s correlation analyses were conducted to investigate the association between the measured stoke volume and the ones calculated with the equations. Moreover, we calculated the effect size and the mean differences (bias) to examine potential differences between the measured SV and the estimated SV. On the basis of these statistical outcomes, we ranked the formulae from best (rank of 1) to worst (rank of 22). To ensure data integrity we removed values for SV that were outside the range of 61–145 mL (Kawel-Boehm et al., 2020), attributing them to movement artefact and blood pressure data that were higher or lower than two standard deviations. Statistical analyses were conducted in Excel spreadsheets (Microsoft Office, Microsoft, Washington, United States of America). All results are presented as mean ± SD unless otherwise stated.

## 3 Results

### 3.1 Comparison of formulae predicting stroke volume

The evaluation of the formulae was based on the comparison of the error of prediction. This residual analysis compared the sum of the squared residuals generated by each formula using the same data set. For this reason the data from two participants had to be excluded for three formulae from de Simone et al. (de Simone et al., 1997), as their equations were created for a normal-weight population and two participants did not conform to this criteria, with their body mass index being higher than 25.25 kg/m^2^.

Table 1 presents the results of all evaluated formulae. Specifically, for each of the 22 formulae it provides the measurements for the combined data (i.e., both positions and all ambient temperatures). Based on the values of the statistical parameters derived for each formula (i.e., correlation, effect size, sum of square residuals, and bias) we ranked the formulae from the best (rank of 1) to the worst (rank of 22). The best predictor was determined by calculating the sum of individual ranks for various analyses such as. For instance, the total ranking for formula 72 was 15, with the correlation rank being 1, effect size rank being 11, sum of square residuals rank being 1, and bias rank being 2.

For the body positions (supine, 60°HUT), and ambient temperatures (25.4°C and 35.4°C) investigated, the formulae providing the best predictions of SV are presented in Table 3. For both body positions (supine and HUT), the best equations for both temperatures was formula 72 while for the thermoneutral condition formula 71 (Starr and Schnabel, 1954) and formula 4 from Jackson (Jackson, 1955) were the most accurate. For heatwave (35.4°C) the formula proposed by Bridwell et al. (Bridwell et al., 1956) was most accurate. For the supine position only, the best formula for all ambient temperatures was formula 68 (Starr and Schnabel, 1954), and for the heat condition both formula 68 (Starr and Schnabel, 1954) and a formula proposed by Starr using the data from Grollman (Grollman, 1930; Starr, 1954). For the 60° HUT position, for all temperature conditions the best formula was proposed by Bridwell et al. (Bridwell et al., 1956).

**TABLE 3:** Comparison of the best formulas for predicting stroke volume (based on the absolute values) across body positions supine and head-up tilt) and environmental temperatures (both thermoneutral and hot environment, thermoneutral environment and hot environment).

FORMULA	Temperature	Position	Average measured	Average predicted	r	g	SSR	Bias
FORMULA 72 (Starr and Schnabel, 1954)	All	Both	76.85 ± 13.05	66.44 ± 9.99	0.65	0.96	6,256.11	0.67
FORMULA 71 (Starr and Schnabel, 1954)/JACKSON (Jackson, 1955)	25.4°C	Both	77.52 ± 12.31	68.22 ± 9.56	0.73	0.91	3405.53	0.26
BRIDWELL (Bridwell et al., 1956)	35.4°C	Both	75.89 ± 14.13	67.57 ± 4.72	0.54	0.99	2,201.51	0.64
FORMULA 68 (Starr and Schnabel, 1954)	All	Supine 0°	84.34 ± 13.97	78.54 ± 9.46	0.75	0.49	2,763.91	0.27
FORMULA 68 (Starr and Schnabel, 1954)	25.4°C	Supine 0°	83.45 ± 12.56	80.07 ± 8.18	0.78	0.32	2045.29	0.35
FORMULA 68 (Starr and Schnabel, 1954)/STARR 1954: DATA FROM GROLLMAN (Starr, 1954)	35.4°C	Supine 0°	86.24 ± 16.87	75.22 ± 11.36	0.67	0.77	718.62	1.92
BRIDWELL (Bridwell et al., 1956)	All	HUT 60°	69.64 ± 6.46	66.82 ± 4.17	0.66	0.61	1,089.13	2.33
BRIDWELL (Bridwell et al., 1956)	25.4°C	HUT 60°	69.77 ± 6.28	66.51 ± 3.89	0.64	0.76	394.04	1.83
BRIDWELL (Bridwell et al., 1956)	35.4°C	HUT 60°	69.51 ± 6.76	67.34 ± 4.58	0.68	0.42	695.09	2.65

Note: All effect sizes and biases are reported as positive values.

**FIGURE 1:**
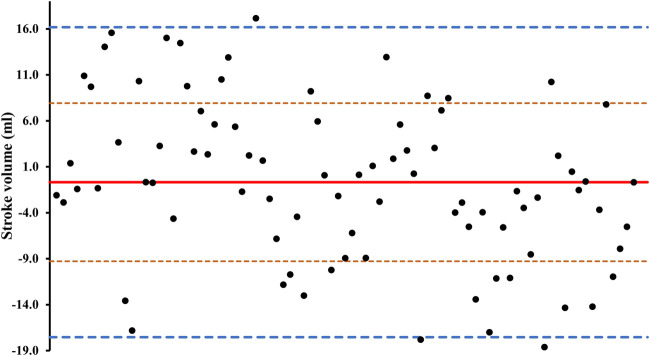
Average differences between simulated and measured stroke volume from formula 72 (Starr and Schnabel, 1954). The solid line represents bias (average difference between the two methods). Fine, orange dashed lines represent the standard deviations. Thick blue dashed lines represent the 95% limits of agreement.

It is evident that the best predictor of SV for both postures and ambient temperatures was formula (equation 72) of Starr et al. (Starr and Schnabel, 1954). The limits of agreement from this formula are plotted in Figure 1. Thus, a 70 kg male would have an average stroke volume of 70 mL (Bruss and Raja, 2024), and the limits of agreement with formula 72 would be from 52.56 to 86.09 with a coefficient of variation between 62.11% and 77.89%.

## 4 Discussion

The present study evaluated the validity/accuracy of formulae predicting stroke volume from ABP, age, heart rate, and a variety of physical characteristics (i.e., mass, height) for supine and upright postures at thermoneutral and hot ambient temperature conditions. Since the original formulae were derived for supine participants (and in some cases cadavers), it is not surprising that the formulae were more accurate predicting SV for the supine position. Predictions were less accurate, but nevertheless reasonable for the upight posture in a hot ambient environment. These formulae could therefore be useful for predicting stroke volume when assessing cardiovascular heat strain of workers conducting work while standing.

Of the 22 formulae evaluated, the best predictors of SV were formulae with the general form:

SV=a+b PP−c DP−d age

where a, b, c, and d, are coefficients derived by regression analyses.

It would be worthwhile to assess the improvement in prediction provided by the inclusion of posture (i.e., supine and upright), ambient temperature and duration of exposure to a particular environment into the equation. From the perspective of wearable technology, information regarding posture could be derived by an accelerometer, ambient temperature with a thermistor, and duration of exposure with a timer. For this development, the empirical data would need to be derived from studies designed specifically for this purpose.

### 4.1 Effect of posture on the prediction of stroke volume

Our data indicated that the best correlations between the predicted and measured SV were observed in the supine position under thermoneutral conditions. This finding is not surprising as most of the existing formulae were either developed or validated in the supine position. More specifically a previous series of studies from Starr and his colleagues created the formulae with measurements on cadavers (Starr and Schnabel, 1954), while another study have employed anesthetized patients during surgery (Skrabal et al., 2005). Furthermore, many studies that were used for validation purposes were conducted with participants in the supine position (Warren et al., 1945; Cathcart et al., 1953). Lastly, in the case of Remington (Remington et al., 1948) the creation of different formulae to estimate SV were based on aortic length measurements taken during autopsies.

### 4.2 Effect of ambient temperature on prediction of stroke volume

It is very important to note that the environmental conditions were not reported in all the aforementioned studies from which the predictive formulae were derived, but were most likely thermoneutral. Formulae derived from supine participants in a thermoneutral environment cannot appropriately predict the SV response during upright posture in a hot environment. During whole-body heating, a vasodilatory response leads to an increase in cutaneous blood flow which decreases ABP, if not matched with a concomitant increase in CO, to compensate for this decrease (Rowell et al., 1969; Crandall et al., 2008). A review by Johnson and Proppe (Johnson and Proppe, 2011) reported that heat exposure (38°C or 46°C and relative humidity 42%) for over an hour leads to no changes in CO (Johnson and Proppe, 2011), however, with longer exposure times, the levels of CO increases as well. Furthermore, the same review reported that during heat exposure, the HR was elevated (Johnson and Proppe, 2011). Another study that presents data from agriculture workers demonstrated that during the 8-h work shift under warm environmental conditions (28.5°C ± 3.3°C) there was an increase in HR, suggesting a moderate-to-high level of work intensity (Ioannou et al., 2019). Additionally, a previous study indicated increased skin and core temperature, and decreased ABP during heat stress compared to normothermia (Gagnon et al., 2016). Finally, our data on mean skin and core temperature that have been previously published (Fisher et al., 2022) indicated that even a small difference in core temperature during the 9-h work shift increased the skin temperature by 2.4 °C during the heatwave (hot environment) compared to pre-heatwave (thermoneutral environment).

These findings indicate the importance of finding an appropriate and easily accessible way to predict the cardiovascular strain of workers during their work shifts when they work in a hot environment or during heatwaves. Thus, we separate our statistical analysis into these three different combinations of temperatures (i.e., both thermoneutral and heatwave conditions, thermoneutral environment and heatwave) and the different positions (both supine and HUT, supine, HUT) and we found that for the hot environment we need a different equation compared to the thermoneutral for the combination of the positions, the supine alone as well as the HUT alone.

### 4.3 Limitations and future studies

The evaluation of the different formulae was based on the data of young adults (range: 19–23). Although the demographics of workers in the manufacturing industry include both younger (18–39 years) and older (40–59 years) workers, younger adults dominate in the computer-related manufacturing industry (Ioannou et al., 2021). It is well known that thermoregulatory function is modified by aging (Anderson et al., 1996). A previous study demonstrated that after 2 hours of exercise with breaks, the capacity of heat dissipation was reduced even in 40-year adults (Larose et al., 2013). Cardiovascular responses are also modified by ageing. Specifically, Minson et al. in their study found that the SV and, in this line, the CO were reduced in older people (age 70 ± 3 years) compared to young (24 ± 1 year) (Minson et al., 1998; Kenney and Munce, 2003). As all participants in this study were young healthy adults, their cardiovascular stress may have been lower than older individuals and/or persons with chronic diseases. As the occurring climate change leads to more frequent and longer heatwaves (Perkins et al., 2012), future studies should validate these findings during heatwaves in real-life conditions with larger populations of healthy and clinical individuals. Finally, our comparisons were performed using equipment that employs the model flow algorithm to calculate SV and not the gold standard methods for measuring SV, thus further investigations are needed for the comparison of these formulae or, even better, the creation of a new formula in healthy participants exposed to different environmental conditions.

## 5 Conclusion

Based on our analysis, it is recommended to measure stroke volume in the supine position and in thermoneutral conditions for better accuracy. However, our results suggest that it is possible to estimate the SV in the upright position as well, using the existing formulae with a good correlation when compared to the SV measured with the Finapres and a relevant small bias. Since the results showed that the SV derived from these formulae is acceptable, it is easy to just measure the blood pressure of a worker during their break and estimate their cardiac strain. As cardiovascular strain is important to be monitored in workers during their work-shift, especially during heatwaves, these equations could be a solution until a more precise method or formula is developed.

## Data Availability

The data that support the findings of this study are available from the corresponding author upon reasonable request.
